# Signal-averaged P wave analysis for delineation of interatrial conduction – Further validation of the method

**DOI:** 10.1186/1471-2261-7-29

**Published:** 2007-10-09

**Authors:** Fredrik Holmqvist, Pyotr G Platonov, Rasmus Havmöller, Jonas Carlson

**Affiliations:** 1 Department of Cardiology, Lund University Hospital, SE 221 85, Lund, Sweden

## Abstract

**Background:**

The study was designed to investigate the effect of different measuring methodologies on the estimation of P wave duration. The recording length required to ensure reproducibility in unfiltered, signal-averaged P wave analysis was also investigated. An algorithm for automated classification was designed and its reproducibility of manual P wave morphology classification investigated.

**Methods:**

Twelve-lead ECG recordings (1 kHz sampling frequency, 0.625 *μ*V resolution) from 131 healthy subjects were used. Orthogonal leads were derived using the inverse Dower transform. Magnification (100 times), baseline filtering (0.5 Hz high-pass and 50 Hz bandstop filters), signal averaging (10 seconds) and bandpass filtering (40–250 Hz) were used to investigate the effect of methodology on the estimated P wave duration. Unfiltered, signal averaged P wave analysis was performed to determine the required recording length (6 minutes to 10 s) and the reproducibility of the P wave morphology classification procedure. Manual classification was carried out by two experts on two separate occasions each. The performance of the automated classification algorithm was evaluated using the joint decision of the two experts (i.e., the consensus of the two experts).

**Results:**

The estimate of the P wave duration increased in each step as a result of magnification, baseline filtering and averaging (100 ± 18 vs. 131 ± 12 ms; P < 0.0001). The estimate of the duration of the bandpass-filtered P wave was dependent on the noise cut-off value: 119 ± 15 ms (0.2 *μ*V), 138 ± 13 ms (0.1 *μ*V) and 143 ± 18 ms (0.05 *μ*V). (P = 0.01 for all comparisons).

The mean errors associated with the P wave morphology parameters were comparable in all segments analysed regardless of recording length (95% limits of agreement within 0 ± 20% (mean ± SD)). The results of the 6-min analyses were comparable to those obtained at the other recording lengths (6 min to 10 s).

The intra-rater classification reproducibility was 96%, while the interrater reproducibility was 94%. The automated classification algorithm agreed with the manual classification in 90% of the cases.

**Conclusion:**

The methodology used has profound effects on the estimation of P wave duration, and the method used must therefore be validated before any inferences can be made about P wave duration. This has implications in the interpretation of multiple studies where P wave duration is assessed, and conclusions with respect to normal values are drawn.

P wave morphology and duration assessed using unfiltered, signal-averaged P wave analysis have high reproducibility, which is unaffected by the length of the recording. In the present study, the performance of the proposed automated classification algorithm, providing total reproducibility, showed excellent agreement with manually defined P wave morphologies.

## Background

Studies on P wave duration and morphology are common in the literature [1-7]. However, the method use for the estimation of P wave duration varies widely [1-7]. As can be expected, the reported estimates of P wave duration vary depending on the underlying heart disease [2-5,7], but more surprisingly, substantial differences between similar study populations can also be found [2,5]. This implies that not only the underlying heart disease but also the methodology may affect the estimation of the P wave duration. To improve comparability between studies, the effects of measurement method on P wave analysis must be investigated. At present, the exact cut-off value defining interatrial conduction delay (presented as a prolongation of the P wave) [8,9] is the subject of much debate [1,6]. However, little attention is paid to the method used.

**Figure 1:**
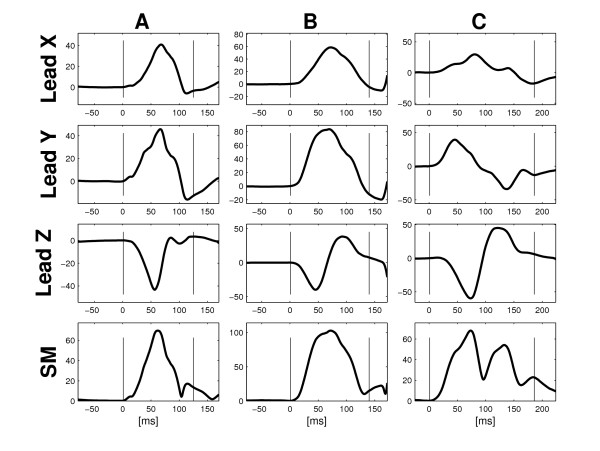
**Different types of P wave morphology**. Typical examples of: Type 1 P wave morphology (A), Type 2 P wave morphology (B) and Type 3 P wave morphology (C). Type 3 morphology has previously been shown to be compatible with Bachmann's bundle block [20,21].

Studies using signal-averaged P wave analysis usually also employ bandpass filtering. However, 'unfiltered' signal-averaged P wave analysis (i.e. signal averaging *without *bandpass filtering) [10] has been shown to reveal differences in P wave morphology between patients with a high prevalence of [2] or propensity for [7] atrial fibrillation and their healthy counterparts. Three different P wave classes (Figure 1), possibly indicating differences in interatrial conduction have been identified [7]. Classification has been performed manually without strictly formalised, objective criteria.

In the present study, the influence of methodology on the estimation of P wave duration was investigated. The requirements for reproducibility using unfiltered, signal-averaged P wave analysis were also investigated, focusing on recording length and automated classification.

## Methods

### Definitions

Agreement is defined as identical results within the accuracy of the measurements. Limits of agreement are used as defined by Bland and Altman [11]. Reproducibility is the term used to express numerical equivalence between results. Total reproducibility refers only to the performance of the automated algorithm.

### Study population and data acquisition

A data set containing ECG data from 131 healthy subjects (denoted the 'evaluation set', mean age 51 ± years, 54% women) without a history of heart disease, was used to investigate the methodological effects on P wave duration and morphological parameters. The same data set was also used to evaluate the automatic P wave classification algorithm.

Another data set, containing ECG data from 107 subjects with a history of heart disease (i.e. atrial fibrillation or hypertrophic cardiomyopathy) was used as a training set for the automated classification algorithm (denoted the 'training set').

The study was approved by the Ethics Committee of Lund University (approval number LU 325-00). Written informed consent was obtained and the study complied with the Declaration of Helsinki. In both data sets, six minutes of 12-lead ECG data had been acquired (1 kHz sampling, 16-bit A/D-conversion, 0.625 *μ*V resolution) using a custom-made, optically isolated PC card (Siemens Elema AB, Solna, Sweden). The data were transferred to a computer and stored for subsequent off-line processing. To enable the analysis of orthogonal P wave morphology, a vectorcardiogram (VCG) was derived from the 12-lead ECG data using the inverse Dower transform [12,13].

### Methods of estimating P wave duration

A subset of the database (48 recordings, representative of the entire database of healthy subjects, in terms of age and gender distribution) was used for this part of the study. P wave duration was manually determined using a dedicated computer program running under MATLAB R14 (The MathWorks Inc., Natick, MA, USA).

Non-magnified ECGs were investigated at 50 mm/s and 10 mV/mm resolution, magnified ECGs at 1000 mm/s and 0.1 mV/mm. Artefact filtering was applied using high-pass filtering (0.5 Hz) to exclude slow baseline drift due to respiratory movement of the thorax, and a 50 Hz bandstop filter to reduce power line interference. Signal averaging (described below) and bandpass filtering (40–250 Hz), with varying noise level cut-off (0.05 *μ*V to 0.20 *μ*V) were applied in the final stages. The combinations of the parameters used (denoted D1 to D8) are summarised in Table 1.

**Table 1:** P wave duration estimation methodologies

	**Magnification**	**Artefact filtering**	**Signal averaging**	**Bandpass filtering**	**Noise threshold**
**D1**	no	no	no	no	-
**D2**	no	yes	no	no	-
**D3**	yes	no	no	no	-
**D4**	yes	yes	no	no	-
**D5**	yes	yes	yes	no	-
**D6**	yes	yes	yes	yes	0.20 *μ*V
**D7**	yes	yes	yes	yes	0.10 *μ*V
**D8**	yes	yes	yes	yes	0.05 *μ*V

Non-magnified ECG (50 mm/s, 10 mV/mm), magnified ECG (1000 mm/s; 0.1 mV/mm). Artefact filtering: high-pass filtering at 0.5 Hz and 50 Hz bandstop filtering. Bandpass filtering: 40–250 Hz. The P wave duration was determined using three different noise level thresholds.

### Signal averaging of P waves

**Figure 2:**
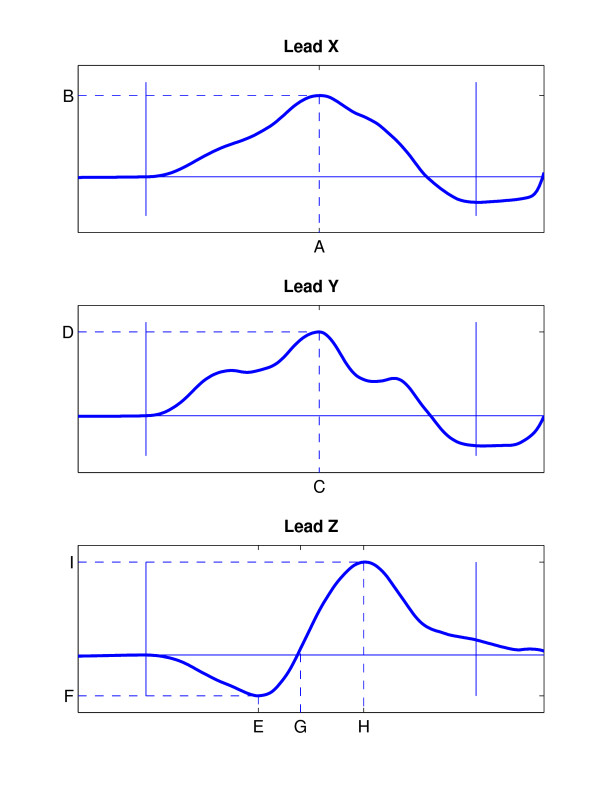
**Measured parameters**. Schematic illustration of the parameters derived from signal-averaged P wave morphologies. Lead X: location (A) and amplitude (B) of maxima; Lead Y: location (C) and amplitude (D) of maxima; Lead Z: location (E) and amplitude (F) of minimum, location of zero-crossing (G), location (H) and amplitude (I) of maximum.

The derived VCG was high-pass filtered (0.5 Hz) to exclude slow baseline drift due to respiratory movement of the thorax. Power line interference was reduced using a 50 Hz bandstop filter. QRS complexes were identified automatically and included according to similarity (a cross-correlation coefficient of *ρ *> 0.9 was applied in order to exclude artefacts and abnormal events such as extra ventricular beats). P waves were extracted using 250-ms signal windows preceding each QRS complex. In cases of unusually long PQ time or P wave duration, the window could be shifted manually in order to fully cover the P wave. Subsequently, the signal windows were time shifted to estimate the maximal correlation in each lead. P waves with a cross-correlation coefficient of *ρ *> 0.9 (in each lead) were grouped together and averaged. The onset and end of the signal-averaged P waves were defined manually. The amplitude at onset was set to 0 V. The method used is described in detail elsewhere [10]. The parameters used to quantify P wave morphology are illustrated schematically in Figure 2.

### P wave morphology classification

P waves were classified into three different types based on their morphology: Type 1 (predominantly positive Leads X and Y and predominantly negative Lead Z), Type 2 (predominantly positive Leads X and Y and biphasic Lead Z (negative, positive) and Type 3 predominantly positive Lead X and biphasic Leads Y (positive, negative) and Z (negative, positive). P waves not classifiable according to these three types were denoted 'Atypical'.

#### Manual classification

Manual P wave morphology classification was performed manually and independently by two expert operators. Each expert classified all P wave morphologies on two occasions, separated by at least three days to estimate intra-rater variability. P wave morphologies assigned different classifications on the first and second occasions were classified on a third occasion.

The classifications of the two experts were then compared to estimate interrater variability. P wave morphologies classified differently by the two experts were finally classified based on a joint decision of the two experts. This "final manual classification" was used when evaluating the automated classification.

#### Automated classification

In order to force the P wave amplitude to 0 V at the end, linear interpolation was applied from the onset to the end of the wave in each lead and the linear segment was subtracted. The positions and amplitudes of the largest maximum and smallest minimum in each lead and the location of the zero-crossings were calculated. If the maximum or minimum had an amplitude less than one fifth of the P wave amplitude it was discarded by the algorithm, thus not influencing the results.

Each lead was described by a three-element vector, {* ; * ; *}. Position one describes the first maximum or minimum (if any) of the P wave (1, -1 or 0). If at least one zero-crossing was located in the mid third of the P wave, position 2 was given the value 1, if not it was given the value 0. Position three describes the second maximum or minimum (if any) of the P wave (1, -1 or 0). The classification of the three different types of P waves and their corresponding vectors are summarised in Table 2. The performance of the automated classification algorithm was evaluated by comparing the results with those of the final manual classification.

**Table 2:** Automated classification algorithm

	**Type 1**	**Type 2**	**Type 3**
**Lead X**	{1 ; * ; 0}	{1 ; * ; 0}	{1 ; * ; 0}
	{1 ; 0 ; *}	{1 ; 0 ; *}	{1 ; 0 ; *}
	{0 ; * ; 1}	{0 ; * ; 1}	{0 ; * ; 1}
**Lead Y**	{1 ; * ; 0}	{1 ; * ; 0}	
	{1 ; 0 ; *}	{1 ; 0 ; *}	{1 ; 1 ; -1}
	{0 ; * ; 1}	{0 ; * ; 1}	
**Lead Z**	{-1 ; * ; 0}		
	{-1 ; 0 ; *}	{-1 ; 1 ; 1}	{-1 ; 1 ; 1}
	{0 ; * ; -1}		

Position one refers to the first peak detected and position three to the second peak (1 = maximum; -1 = minimum; 0 = neither). Position two refers to zero-crossings in the mid third of the P wave (1 = present; 0 = absent). A * indicates that any value is acceptable.

#### Material

Both the data sets (evaluation and training sets) were manually classified as described above. The automated classification algorithm parameters were optimised using the training set and the performance was then evaluated using the evaluation set.

### Recording length

A subset of the database (48 recordings) was used for this part of the study. Data were analysed with respect to standard P wave morphology parameters using 6-min (L1), 3-min (L2), 1-min (L3), 30-s (L4) and 10-s (L5) segments. In an additional analysis (L6) a 10-s segment with a lower signal resolution (sampling frequency 500 Hz, sampling resolution 5 *μ*V) was applied. The P wave morphology parameters from (L1-L6) were compared with a baseline 6-min analysis (as described above) to assess the agreement between the clinical measurements.

### Statistical analysis

Data are presented as the mean and the standard deviation. Paired data were compared using the Wilcoxon matched-pairs test. The chi-squared test was used to evaluate the relationship between dichotomous variables. Statistical significance was defined as P < 0.05. The Bland-Altman method [11]was used to assess the agreement between samples. All statistical analyses were performed using STATISTICA for Windows version 6.1 (StatSoft, Inc., Tulsa, OK, USA).

## Results

### P wave duration

**Figure 3:**
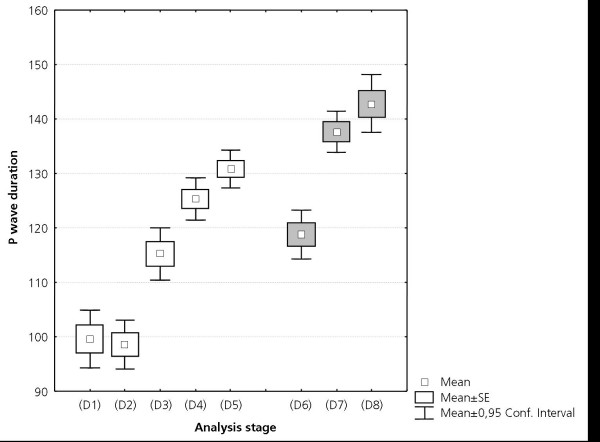
**P wave duration and methodology**. Estimates of average P wave duration in the study population using different methodologies. The various combinations of parameters (D1 to D8) are summarised in Table 1. The three shaded boxes (D6-D8) represent estimates from bandpass-filtered analysis.

The P wave durations measured using the first five combination of parameters were 100 ± 18 ms (D1), 99 ± 16 ms (D2), 115 ± 17 ms (D3), 125 ± 13 ms (D4) and 131 ± 12 ms (D5). The differences were significant (P < 0.0001) for all comparisons with the exception of D1 vs. D2 (P = 0.7). These results are illustrated in Figure 3.

The P wave durations in parameter combinations D6, D7 and D8 were 119 ± 15 ms, 138 ± 13 ms, and 143 ± 18 ms, respectively. These differences were also significant (P < 0.01 for all comparisons). Moreover, the estimates of P wave duration using these parameter combinations were all significantly different from the estimate obtained with D5 (P < 0.01 for all comparisons).

### P wave morphology classification

The intra-rater agreement was 96% for both experts while the interrater agreement was 94%. The final manual classification of the evaluation set, resulted in the following distribution of P wave morphologies: 35/63/0/2 % for Type 1, Type 2, Type 3 and Atypical, respectively.

The optimised classification criteria resulted in correct classification in 97 of the 107 subjects in the training set (91%). The automated classification by the algorithm agreed in 90% of the cases with the final manual classification of the evaluation set. The distribution of P wave morphologies was 39/59/1/2 % for Type 1, Type 2, Type 3 and Atypical, respectively. The difference in distribution of Types 1 and 2 was not statistically significant compared with the final manual classification (P = 0.56). Differences in the distribution of Type 3 and Atypical were not analysed due to the small numbers.

### Recording length

**Figure 4:**
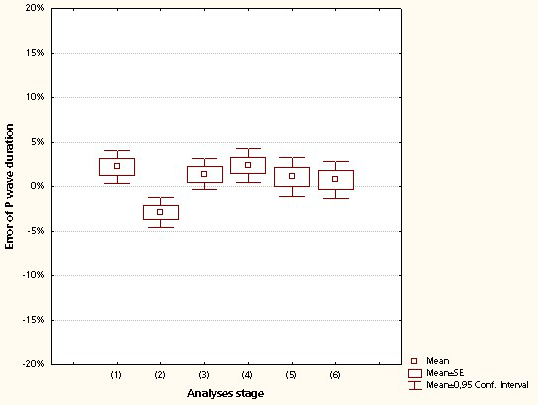
**Median error in P wave duration as a result of recording length**. Median error (%) in the P wave duration estimate as a result of recording length. L1 = 6 min; L2 = 3 min; L3 = 1 min; L4 = 30 s; L5 = 10 s, and L6 = 10 s recording length with lower sampling resolution (see text).

The mean error in all parameters was similar in all the segments analysed regardless of recording length (95% limits of agreement within 0 ± 20% for all parameters and segments). Individual parameters and their corresponding limits of agreement are listed in Table 3. The results of the P wave duration analyses are illustrated in Figure 4.

**Table 3:** Recording length

	**6 min**	**3 min**	**1 min**	**30 s**	**10 s**	**10 s LR**
**P dur**	2.9 ± 18	-3.7 ± 15	1.9 ± 16	3.2 ± 17	1.4 ± 19	1.0 ± 18
**Xmax pos**	0.1 ± 4.7	-1.0 ± 5.8	0.6 ± 15	0.7 ± 13	0.8 ± 18	1.2 ± 18
**Xmax amp**	0.0 ± 0.4	0.0 ± 1.9	-0.1 ± 4.2	-0.2 ± 4.5	0.1 ± 5.6	-0.1 ± 5.7
**Ymax pos**	0.1 ± 4.8	-1.5 ± 8.6	-1.0 ± 7.7	-0.6 ± 10	-1.4 ± 11	-0.9 ± 11
**Ymax amp**	0.0 ± 0.6	-0.4 ± 4.7	-1.3 ± 13	-0.8 ± 14	0.2 ± 18	0.0 ± 17
**Zmin pos**	0.1 ± 4.7	-0.9 ± 5.7	0.6 ± 9.3	-0.2 ± 9.0	-1.6 ± 12	-1.1 ± 11
**Zmin amp**	0.1 ± 0.7	-0.1 ± 2.0	0.4 ± 5.0	0.3 ± 4.1	0.7 ± 6.1	0.9 ± 6.2
**Zzero pos**	0.0 ± 0.4	-1.0 ± 5.5	0.9 ± 9.2	0.1 ± 8.8	-1.5 ± 12	-0.7 ± 12
**Zmax pos**	0.1 ± 21	-4.6 ± 22	-2.3 ± 23	-2.2 ± 27	-1.0 ± 31	-1.8 ± 29
**Zmax amp**	0.1 ± 3.1	-0.7 ± 4.3	-1.7 ± 14	-0.7 ± 7.4	0.7 ± 12	0.1 ± 12
**Nadir pos**	-0.6 ± 10.2	-2.5 ± 17	-0.5 ± 12	-0.4 ± 12	-1.0 ± 17	1.9 ± 27
**Classification**	98%	100%	96%	96%	98%	98%

Median error, in percentage, and corresponding 95% limits of agreement (derived from the Bland-Altman analysis) of the measured P wave morphology parameters, as a function of the recording length. The row **Classification **gives the percentage correctly classified. All comparisons were made using a standard 6-min recording. (LR = low signal resolution)

The results of the 6-min analyses (L1) were comparable to those with shorter recording lengths (L2-L5). The analysis revealed no considerable error resulting from lower signal resolution (L6) (Table 3). The performance of the automated classification algorithm was thus not affected by the length of the recording, showing only occasional changes in classification between the different recording lengths (Table 3).

## Discussion

### P wave duration

The methods used for P wave analysis vary greatly, as do the reported P wave durations in various patient populations [3-5]. The present study is, to the best of our knowledge, the first attempt to compare these different methodologies. The marked differences in P wave duration resulting from the method used underscores the need for method validation and uniformity in order to increase comparability between studies. The ongoing debate on the appropriate cut-off value for interatrial conduction delay [1,6], seems to be inappropriate bearing in mind the results of the current study.

Few invasive studies report average total atrial activation time, but when reported it has been shown to be approaching 120 ms [14,15]. In other invasive studies where right and/or left atrial activation time are reported separately, these commonly exceed 65 to 75 ms [16-18]. There is thus evidence that the 'true' P wave duration on average exceeds 120 ms in invasive measurements in a wide variety of study populations. Moreover, in one study reporting invasively measured biatrial activation time, as well as P wave duration estimated from conventional ECG, the former was 20% longer than the latter [14]. All these findings imply that the longer estimates of P wave duration, observed in the present study when magnification, filtering and averaging are applied, are the result of better delineation of the P wave as the noise level decreases. Interestingly, when 15 ms was added to the widely accepted cut-off value for interatrial conduction delay (120 ms), the prevalence of interatrial block in the entire study population was equal to that expected based on population age distribution [19].

**Figure 5:**
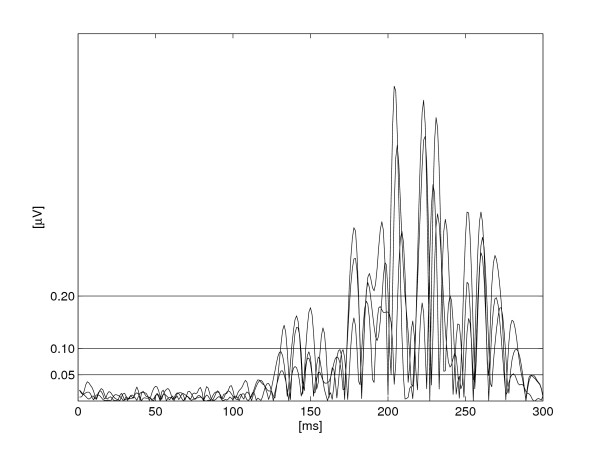
**Bandpassed-filtered signal-averaged P wave analysis**. A representative example of a bandpass-filtered (40–250 Hz), signal-averaged P wave in which there is an evident dependency between the noise threshold used and the estimate of the P wave duration.

The estimates of P wave duration obtained when using the widespread technique of bandpass filtering of the signal-averaged P waves, varied considerably depending on the noise cut-off value chosen (Figure 5). This has important implications when comparing results from different studies applying this technique.

### P wave morphology classification

Based on findings in invasive studies it is reasonable to assume that the right and left atrial activation times are about the same [16-18]. It is therefore logical to assume that the first and the last third of the P wave primarily represents right and left atrial activation respectively, whereas the middle third is likely to represent electrical activity from both atria and interatrial septum. This assumption is supported by invasive studies [14]. Therefore, although the cut-off value was arbitrarily chosen to optimise the performance in the training set, the cut-off values used in the classification algorithm are based on scientific findings.

In two previous studies carried out by our group it has been shown that analysis of P wave morphology may reveal differences that would not have been detectable had standard methods been used [2,7]. It is theoretically plausible that these changes are the result of different preferential interatrial conduction routes in these subjects [7]. Preliminary, yet unpublished, data from invasive studies support this hypothesis. The present study clearly shows that, although not based on formalised criteria, the manual classification of P wave morphology is highly reproducible, with low intra- and interrater variability. The results of the classification algorithm are naturally totally reproducible as they are based on strict criteria. The marginally poorer agreement between the automated and the final manual classification, compared with the intra- and interrater agreement in manual classification, may be the price one has to pay to ensure reproducibility.

### Recording length

In the present study there were no signs of declining performance as a result of shortening the recording time, even when considering only the recordings with the fewest averaged P waves (i.e. three to five). The method was also robust when recordings with lower sampling resolution were analysed. This has potentially important implications since many commercially available ECG storing systems use this sampling resolution. The demonstrated robustness, together with the sufficient recording length of ten seconds, allows the use of ECGs stored using such systems, at least if the ECGs are of sufficiently 'high' quality.

### Study limitations

All the ECG recordings used in the present study were of high quality. Therefore, no inferences regarding the performance of the method on ECGs of poor quality can be made.

## Conclusion

The present study illustrates a marked difference in P wave duration depending on the methodology employed, thus explaining differences in absolute values reported by different groups. It is therefore necessary to define each method carefully or to recommend a universal method of estimating P wave duration.

The automated analysis of P wave duration and morphology demonstrated in this study has high reproducibility and is not affected by the length of the recording. As short as ten-second-long ECGs recorded and stored using commercially available systems with lower signal resolution can be used for non-invasive studies of interatrial conduction. The proposed automated classification algorithm also showed excellent agreement with manually defined P wave morphologies.

## Competing interests

The author(s) declare that they have no competing interests.

## Authors' contributions

FH designed the study, included patients, analysed and interpreted the data and drafted the manuscript. PGP interpreted data and revised the manuscript. RH included patients, analysed and interpreted data and revised the manuscript. JC co-designed the study, analysed and interpreted data and revised the manuscript. All authors read and approved the final manuscript.

## Pre-publication history

The pre-publication history for this paper can be accessed here:


